# Late Giant Aortic Root Aneurysm Following Aortic Valve Replacement

**DOI:** 10.14797/mdcvj.1118

**Published:** 2022-07-20

**Authors:** Rabia Azam, Metesh Acharya, Sonam Vadera, William Adair, Giovanni Mariscalco

**Affiliations:** 1 Glenfield Hospital, Leicester, UK

**Keywords:** aortic root aneurysm, aortic valve replacement, aortic root expansion

## Abstract

A 74-year-old female with previous permanent pacemaker insertion for complete heart block and no history of connective tissue disease presented to our regional cardiothoracic center with progressive exertional shortness of breath. Nine years later, when the patient was 83 years old, a computed tomography scan of the thoracic aorta revealed an isolated aneurysm of the aortic root measuring 7.6 × 5.1 cm at the sinus of Valsalva.

A 74-year-old female with previous permanent pacemaker insertion for complete heart block and no history of connective tissue disease presented to our regional cardiothoracic center with progressive exertional shortness of breath. Transthoracic echocardiogram (TTE) confirmed severe calcific aortic stenosis associated with a pseudo-bicuspid aortic valve, and coronary angiography (Figure 1 A) showed an aortic root measuring within the normal diameter range. She underwent urgent aortic valve replacement using a 23-mm Aspire porcine bioprosthesis. Intraoperative transesophageal echocardiography (TEE) demonstrated a well-functioning aortic valve replacement, and she was discharged home after an uncomplicated recovery.

**Figure 1:**
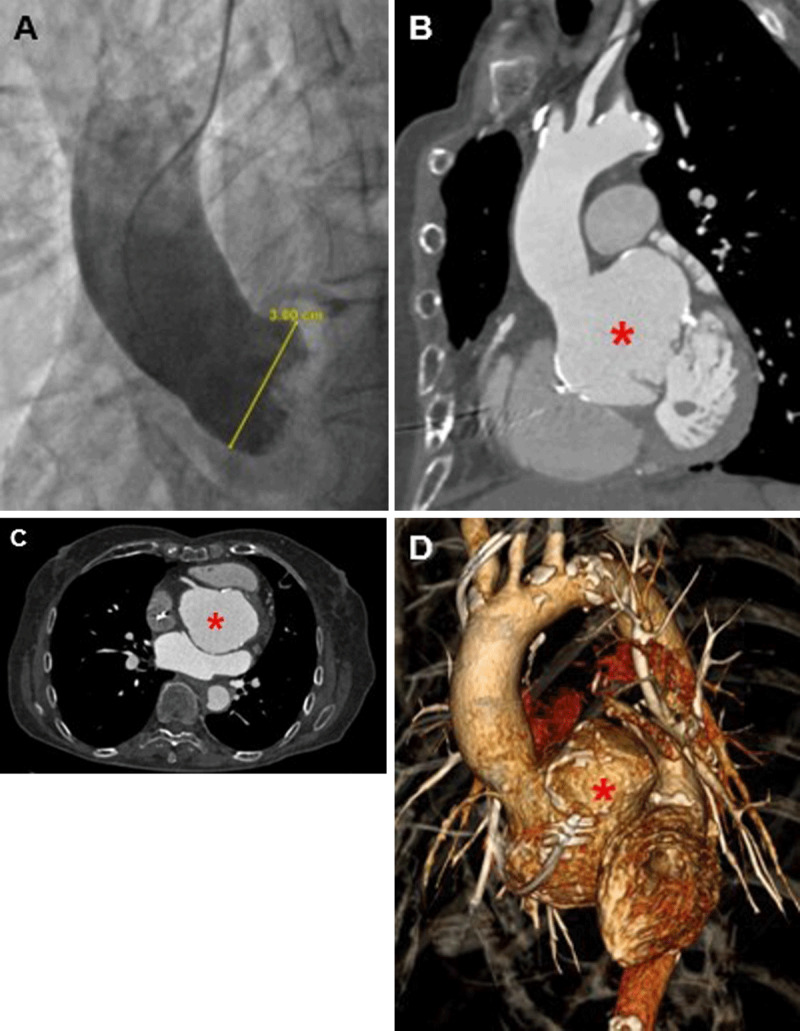
**(A)** Preoperative invasive angiography demonstrates an aortic root of normal diameter. **(B, C)** Computed tomography (CT) and **(D)** 3-dimensional reconstruction of CT scan performed 9 years after bioprosthetic aortic valve replacement shows a giant isolated aortic root aneurysm (red asterisk).

Nine years later, when the patient was 83 years old, a surveillance transthoracic echocardiogram suggested aortic dilatation. A computed tomography (CT) scan (Figure 1 B-D) of the thoracic aorta was performed for further evaluation, revealing an isolated aneurysm of the aortic root measuring 7.6 × 5.1 cm at the sinus of Valsalva, with normal dimensions of the sinotubular junction, ascending aorta, and distal thoracic aorta. This unusual pattern of aortic dilatation raised the possibility of syphilitic aortitis, although the patient’s history could not corroborate the CT findings.

The case was discussed at our departmental aortic multidisciplinary team meeting. Owing to the patient’s generalized frailty and the perceived perioperative risks associated with repeat surgery, conservative management was advised with ongoing medical follow-up.

Judicious clinical assessment combined with periodic echocardiographic surveillance following surgical valve intervention is essential to monitor residual or concurrent non-intervened valve disease. As demonstrated in this case, such an approach can also identify novel pathologies affecting the heart and great vessels, permitting their operative management where appropriate. Although the delayed aortic root expansion following aortic valve replacement is well documented, the prophylactic replacement of the aortic root when not significantly dilated (< 45 mm) is not justified by current guidelines in non-aortopathy populations.

